# An artificial neural network-based radiomics model for predicting the radiotherapy response of advanced esophageal squamous cell carcinoma patients: a multicenter study

**DOI:** 10.1038/s41598-023-35556-z

**Published:** 2023-05-29

**Authors:** Yuchen Xie, Qiang Liu, Chao Ji, Yuchen Sun, Shuliang Zhang, Mingyu Hua, Xueting Liu, Shupei Pan, Weibin Hu, Yanfang Ma, Ying Wang, Xiaozhi Zhang

**Affiliations:** 1 Department of Radiation Oncology, The First Affiliated Hospital of Xi’an Jiaotong University, Xi’an, China; 2 Department of Computer Science and Communications Engineering, Graduate School of Fundamental Science and Engineering, Waseda University, Tokyo, Japan; 3 Department of Radiation Oncology, The Second Affiliated Hospital of Xi’an Jiaotong University, Xi’an, China

**Keywords:** Cancer, Computational biology and bioinformatics

## Abstract

Radiotherapy benefits patients with advanced esophageal squamous cell carcinoma (ESCC) in terms of symptom relief and long-term survival. In contrast, a substantial proportion of ESCC patients have not benefited from radiotherapy. This study aimed to establish and validate an artificial neural network-based radiomics model for the pretreatment prediction of the radiotherapy response of advanced ESCC by using integrated data combined with feasible baseline characteristics of computed tomography. A total of 248 patients with advanced ESCC who underwent baseline CT and received radiotherapy were enrolled in this study and were analyzed by two types of radiomics models, machine learning and deep learning. As a result, the Att. Resnet50 pretrained network model indicated superior performance, with AUCs of 0.876, 0.802 and 0.732 in the training, internal validation, and external validation cohorts, respectively. Similarly, our Att. Resnet50 pretrained network model showed excellent calibration and significant clinical benefit according to the C index and decision curve analysis. Herein, a novel pretreatment radiomics model was established based on deep learning methods and could be used for radiotherapy response prediction in advanced ESCC patients, thus providing reliable evidence for therapeutic decision-making.

## Introduction

Esophageal cancer (EC) is one of the fatal subtypes of malignant tumors and has the seventh-highest mortality rate among all subtypes^1^. For Asia, squamous cell carcinoma is the primary pathological subtype of EC. Radical surgery and chemoradiotherapy are crucial treatments for esophageal squamous cell carcinoma (ESCC) patients^2^. Radical radiotherapy is recommended as a preferred treatment for cervical and middle thoracic esophageal carcinoma located at a higher position that is difficult to completely resect by surgery. For unresectable advanced ESCC, chemotherapy and radiotherapy are still needed to relieve symptoms and extend survival^3–5^.

Nevertheless, sensitivity to radiotherapy varies among different patients^6^, leading to significant differences in treatment response. Adverse events and side effects are more likely to be observed in patients with radiation-resistant ESCC^7,8^. To this end, a practical and noninvasive approach that can estimate radiotherapy precisely before treatment implementation needs to be explored in ESCC patients.

In recent decades, the general classification of esophageal contrast (medullary type, fungating type, constrictive type, and ulcerative type) to predict the radiotherapy response has been widely used in clinical work^9,10^. However, this prediction is entirely based on empirical evaluation by radiologists, which causes differences among actual treatment responses. Otherwise, the molecular biomarkers related to radiotherapy sensitivity have not been prospectively validated for routine clinical usage. Recent studies have indicated that radiomics based on artificial intelligence (AI) can extract noninvasive radiographic virtual biopsy biomarkers, effectively providing predictive information for treatment response^11,12^. Lu et al.^13^ found that the deep learning-based model showed high accuracy in identifying the origins of cancers of unknown primary. Zhong^14^ indicated that multiparametric magnetic resonance imaging (mp-MRI)-based radiomics features could be considered prognostic factors in patients with localized prostate cancer after radiotherapy. Gao^15^ showed that radiomics signatures based on longitudinal diffusion-weighted MRIs could be used to estimate radiotherapy effects preoperatively. Zhu^16^ reported that a nomogram model based on computed tomography (CT) imaging radiomic signatures and clinical factors showed proper sensitivity and specificity in estimating the risk of local recurrence in nasopharyngeal carcinoma (NPC) after intensity-modulated radiotherapy (IMRT).

Previous radiomics studies reported that radiomics features significantly improved the evaluation of the complete pathological response after neoadjuvant chemoradiation in EC patients^17^. However, few relevant studies based on radiomics to predict the response of radiotherapy in ESCC have been reported. Herein, in this study, a large cohort of 248 patients with ESCC was used to develop a novel baseline CT-based radiomics signature model by a deep learning algorithm to validate their performance in predicting response to radiotherapy.

## Materials and methods

### Patients

Baseline information and imaging data, including demographic data, clinical data, pathological findings of biopsies before treatment, pre- and postsurgical imaging data, and surgical records, of patients with ESCC who underwent radiotherapy at Institution 1 (The First Affiliated Hospital of Xi'an Jiaotong University) from 2013 to 2019 were collected and analyzed. Moreover, we also retrieved and collected the same related data from patients with ESCC who received radiotherapy at Institution 2 (The Second Affiliated Hospital of Xi'an Jiaotong University) from 2017 to 2019. All patients underwent CT examination at the time of positioning before the beginning of their radiotherapy, and the CT data were collected retrospectively from 2021 to 2022. The main inclusion criteria were as follows: (1) biopsy-diagnosed ESCC; (2) clinically diagnosed advanced ESCC by CT and contrast imaging; (3) underwent complete radical radiotherapy (and did not drop out during the treatment); and (4) pre- and postradiotherapy imaging data were recorded after the same institution. The main exclusion criteria were as follows: (1) no biopsy or pathological confirmation; (2) no surgery or more than two weeks of adjuvant chemotherapy before radiotherapy; and (3) no reexamination of imaging data after radiotherapy. The general classifications of all patients were based on the Chinese CSCO guidelines^18^. We used the standard of clinical staging for nonoperative esophageal cancer (Draft) for tumor grading^19^. Written informed consent was obtained from all patients in this study. All methods undertaken in this work were carried out in accordance with the relevant guidelines and regulations. All patients' clinical information was consecutively enrolled, and the Ethics Committee of Xi'an Jiaotong University approved this study.

**Figure 1:**
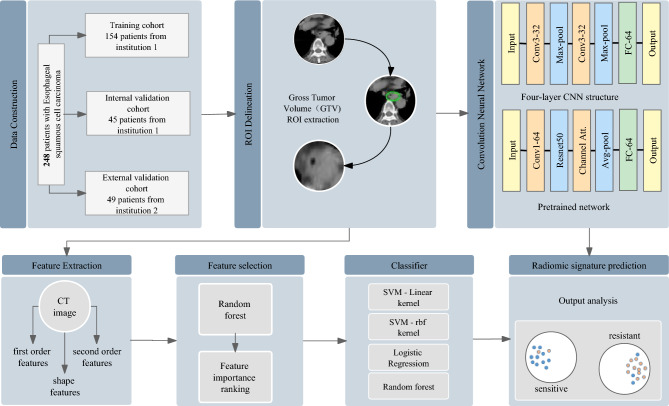
Flowchart illustrating the steps for developing the radiomic sensitivity prediction model, which includes patient categorization, tumor volume ROI delineation, model selection and output analysis. This flow chart shows the superiority of the neural network model in the end-to-end aspect. The prediction results can be obtained directly without the tedious feature extraction steps of machine learning. In practical applications, the generalization performance of the neural network model is also better.

Twenty percent of the patients from Institution 1 were randomly selected for the internal validation cohort, and the rest of the patients from Institution 1 were grouped into the training cohort. The patients from Institution 2 were chosen as the external validation cohort. The detailed experimental flow is illustrated in Fig. 1.

### Radiotherapy treatment and response assessment

All patients in our research accepted the localizing by Brilliance CT locator (Philps, UK) before radiotherapy. The main operating parameters: tube voltage: 140 kV; tube electric current: 500 mA; beam spacing: 0.625 layer thickness; rotation time: 0.5 s; matrix: 512 × 512; detector size: 24 mm. The scanning parameters: layer thickness ≤ 5 mm; interval ≤ 5 mm.

Before the scanning of CT, two radiotherapy technologists and doctors were required to accompany with the patients. All patients were required to fast for 4 to 6 h before CT scanning and drink 0.5 L of water during the scanning to make the esophagus dilated as much as possible. All patients were asked to hold their breath during using the multi-layer spiral CT machine to continuously scan the chest. The scanning range started from the upper edge of the supraclavicular fossa 5 cm above, down to the level of the 1st lumbar vertebra. Then the data of CT scanning was passed to the workstation for reconstruction.

All patients in this study received radical radiotherapy, with a dose of radiation ranging from 60 to 66 Gy at an energy of 6 MV, 1.8–2.0 Gy/fraction, 5 times per week. Organs at risk (OAR), including the bilateral lungs, spinal cord, gastric duct and heart, were outlined for protection. The maximum tolerated doses for key normal structures were as follows: spinal cord: < 40 Gy; heart V40 ≤ 30%; bilateral lungs: V20 ≤ 28% and V30 ≤ 20%; and stomach: V40 ≤ 40%. However, because the data were retrieved retrospectively, the actual dose of radiotherapy for each patient was slightly different. Despite this, each radiotherapy plan fell within the scope of the radical dose recommended by the NCCN guidelines^20^.

Imaging specialists assessed patients' imaging data to measure the tumor's maximum diameter in each plane recommended by RECIST 1.1 guidelines^21^. Then, the maximum diameter shrinkage rate of the tumor could be calculated (preradiotherapy maximum diameter/postradiotherapy maximum diameter), which is popularly used in clinical work to evaluate the treatment response. According to the maximum tumor diameter shrinkage rate for all patients, we selected 0.5 as the potential threshold to divide patients into two categories. After experiments, we chose the optimal cutoff value, which was 0.5 as the threshold to divide patients (tumor reduction rate greater than 50% and tumor reduction rate less than 50%).

### Delineation of regions of interest (ROIs)

The continuous planes of CT images in all patients were outlined by two radiologists with more than eight years of experience in radiotherapy target area delineation using Monaco 5.2. Furthermore, another radiologist with more than 15 years of experience checked the target area and obtained the final radiotherapy target area, which guaranteed that the whole tumor of each patient was reflected by the target area from continuous planes of CT images. In this trial, we used the outlined target area images for the construction of a radiotherapy response model. To minimize the influence of the tumor margin on the model, for each patient, we chose the images that best described the tumor site.

### Dataset pre-processing

The patient CT and target delineation data collected in this experiment are stored in DICOM file format, and the CT data in the DICOM files are converted into 2D PNG format images through Python. In this way, we can extract 10 to 60 slices of 2D images outlining the tumor target area from each patient. Afterwards, professional radiologists selects the most representative 2D CT images of the tumor (usually images around the maximum diameter of the tumor). The dataset collected from institution 1 was randomly divided into training set validation sets on a patient basis. The data collected from Institution 2 is distributed to the external validation group.

In machine learning experiments, 2D CT images are transformed into feature vectors through feature extraction and subsequent experiments are conducted. In artificial neural networks, we applied data augmentation methods such as rotation, flipping, zooming, and distortion to the CT images. Through data augmentation, the dataset of CT images increased to 6000. And the 2D CT images were the direct input to the artificial neural network models.

### Prediction using machine learning

In this study, patients from institution one (training cohort and internal cohort) were used to construct and verify the classification model. Radiomic features of the CT images were extracted using PyRadiomics image extraction software version 3.0. A total of 102 2D features were extracted from each patient, including first-order, shape 2D, gray level cooccurrence matrix (GLCM), gray level run length matrix (GLRLM), gray level size zone matrix (GLSZM), neighboring gray-tone difference matrix (NGTDM), and gray level dependence matrix (GLDM) features^22^. Moreover, we grouped the correlated features (> 0.8) by the Pearson correlation coefficient algorithm, and the less predictive features in the same group were ignored in the feature selection algorithm (Supplementary Fig. S1). The random forest algorithm was used to decrease the data dimensions and select the most predictive features^23^. The formula and explanation of the feature selection algorithm are as follows:

1 $${\text{Importance}}\left( {\text{X}} \right) = {\raise0.7ex\hbox{${\sum\nolimits_{{{\text{i}} = 1}}^{{\text{N}}} {\left( {{\text{err}}_{{{\text{OOB2}}}} - {\text{err}}_{{{\text{OOB1}}}} } \right)} }$} \!\mathord{\left/ {\vphantom {{\sum\nolimits_{{{\text{i}} = 1}}^{{\text{N}}} {\left( {{\text{err}}_{{{\text{OOB2}}}} - {\text{err}}_{{{\text{OOB1}}}} } \right)} } N}}\right.\kern-0pt} \!\lower0.7ex\hbox{$N$}}$$

in which X represents the feature, ${\mathrm{err}}_{\mathrm{OOB}2}$ represents the out-of-bag error when we add noise to feature X and ${\mathrm{err}}_{\mathrm{OOB}1}$ represents the out-of-bag error without adding noise. Ten sets of experiments with the number of features ranging from one to ten were applied to determine the optimal number of features for the model. Hence, the five most predictive features were selected to train the classifier and achieved the best performance. Meanwhile, multiple classifiers were tested in this study, including support vector machine (SVM) with linear kernel, SVM with radial basis kernel linear regression model^24,25^, linear regression model^26^ and random forest. After comparing the AUCs of the classifiers, we selected the random forest algorithm because it outperformed all other classifiers.

### Prediction using deep learning

In this study, patients from institution one were used to build the training cohort and internal validation cohort, and patients from institution two were used to build the external validation cohort to verify the efficiency of generalization.

The CNN model has been proven to be very effective in the field of image classification^27–29^. End-to-end CNN models provide precise prediction results without additional image feature extractions, which significantly improves the model's efficiency. In this study, multiple neural networks were used to classify the CT images of the patients. The model's learning rate was set to 0.0005, and the root mean square prop (RMSprop) optimizer was used. Meanwhile, we used binary cross entropy as the loss function of the mode; the formula is as follows:

2 $$H_{p} \left( q \right) = - \frac{1}{N}\sum\nolimits_{i = 1}^{N} {y_{i} \cdot \log \left( {p\left( {y_{i} } \right)} \right) + \left( {1 - y_{i} } \right) \cdot \log \left( {1 - p\left( {y_{i} } \right)} \right)}$$

in which *y*_*i*_ represents the label for each image, $\mathrm{p}({\mathrm{y}}_{\mathrm{i}})$ represents the probability of the image being positive and $\mathrm{q}$ represents the real distribution. In addition, to prevent overfitting problems, we applied a dropout layer and set the dropout rate to 0.2 (randomly ignoring 20% of the neurons). Finally, a sigmoid layer was applied to the model before the output layer to normalize the outputs. The sigmoid function is defined as follows:

3 $$S\left({x}_{i}\right)=\frac{1}{1+{e}^{-{x}_{i}}}.$$

**Figure 2:**
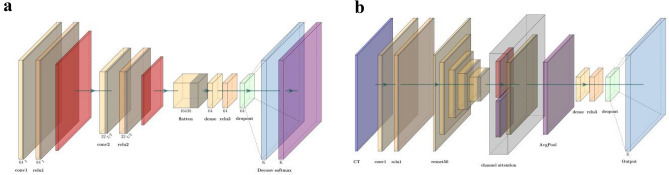
(**a**) CNN model consisting of two convolution layers with a kernel size of 3*3. Each convolutional layer follows the ReLU and max-pooling functions. The dropout function can significantly prevent the overfitting problems of the model. (**b**) The pretrained model was mainly built with pretrained ResNet50 from ImageNet, and the channel attention layer used both max pooling and average pooling to calculate the channel attention feature map.

The batch size was set to 16 and achieved the best performance among other sizes. By training the neural network model, the probability statistics of patients' radiotherapy sensitivity were finally obtained (a probability of more than 0.5 is sensitive, and a probability of less than 0.5 is resistant). The detailed structure of the neural network models is illustrated in Fig. 2a.

Diverse research on pretrained neural networks has shown state-of-the-art performance in image classification tasks^30,31^. Meanwhile, the channel attention mechanism has been proven to be efficient in improving the performance of deep learning models. In this study, a pretrained ResNet50 (trained on ImageNet from Keras) with a channel attention layer was applied^32–34^. The channel attention layer was applied to capture the most critical channels from the model's output; the formula of the function is as follows:

4 $${M}_{c}\left(F\right)= \sigma \left(MLP\left(AvgPool\left(F\right)\right)\right)+MLP\left(MaxPool\left(F\right)\right),$$

in which $\mathrm{MLP}$ represents the multilayer perceptron and $\mathrm{F}$ represents the inputs. The learning rate was set to 0.01, and the Adam optimizer was used. The loss function in these experiments was also binary cross entropy. Meanwhile, the dropout rate of the dropout layer was set to 0.5. After multiple experiments, the batch size was set to 32 and achieved the best performance. The pretrained model outperformed all the other methods we applied in this study. The optimal classifier with the best AUC was used for further exploration. The detailed structure of the neural network models is illustrated in Fig. 2b.

When evaluating the predictive performance of each models, we use the area under the ROC curve (AUC value) to measure the accuracy of each predictive models. However, the area under the ROC curve can not take into account the clinical practicability of the prediction model. Therefore, we use the decision curve to further evaluate each models. The decision curve integrates the preferences of decision makers into the analysis, and can actually evaluate the benefit in clinical practice after using this method. Thus, it meets the actual needs of clinical decision-making and is increasingly widely used in clinical analysis^35^

### Statistical analysis

We used SPSS statistical software version 18 to calculate the significant differences by the X2 test or Fisher’s exact test for categorical variables. A 2-tailed *P* < 0.05 was considered statistically significant. The association analysis of univariate logistic was also calculated by SPSS statistical software version 18. The calibration curve and decision curve were performed to test the calibration performance and clinical utility^35^. Python software version 3.8 (Python) was used for graphic depiction.

## Results

### The baseline clinical characteristics of patients and association analysis with radiotherapy

Our study enrolled 248 ESCC patients, including 154 (62.1%) patients in the training cohort, 45 (18.1%) in the internal validation cohort and 49 (19.8%) in the external validation cohort. The clinical characteristics of patients in the three cohorts are summarized in Table 1. The mean (SD) ages of the training, internal validation, and external validation cohorts were 69.36. The entire cohort comprised 245 patients (98.8%) who were diagnosed with clinical stage III ESCC at the time of treatment. The training cohort comprised 62 responders and 92 nonresponders, the internal validation cohort comprised 14 responders and 31 nonresponders, and the external validation cohort comprised 18 responders and 31 nonresponders. There were no statistically significant differences in the sex ratio, clinical stage, tumor location, general type, or tobacco use during radiotherapy between responders and nonresponders in the training, internal validation, and external validation cohorts (Table 1).

**Table 1:** Patient characteristics in the training, internal validation and external validation cohorts.

	Institution 1						Institution 2		
	Training cohort, NO. (%)			Internal validation cohort, NO. (%)			External validation cohort, NO. (%)		
Characteristic	Respondents n = 62 (40.26)	Nonrespondents n = 92 (59.74)	*P* value	Respondents n = 14 (31.11)	nonrespondentsn = 31 (68.89)	*P* value	Respondents n = 18 (36.73)	nonrespondentsn = 31 (63.27)	*P* value
Sex									
Female	17 (27)	21 (23)	0.52	4 (29)	12 (39)	0.75	4 (22)	5 (16)	0.88
Male	45 (63)	71 (77)		10 (71)	19 (61)		14 (78)	26 (84)	
Age, mean (SD),y	69.90 (7.69)	69.10 (9.90)	0.65	68.71 (7.89)	68.68 (11.00)	0.61	70.44 (11.50)	69.39 (9.14)	0.72
Regression rate (SD),%	70.07 (12.78)	24.84 (22.94)	0	67.29 (12.36)	12.19 (53.15)	0	63.21 (10.15)	9.89 (54.23)	0
Clinical stage									
1	1 (2)	0 (0)	0.83	0 (0)	1 (3)	0.64	1 (5)	3 (10)	0.09
2	10 (16)	20 (22)		3 (21)	1 (3)		2 (11)	10 (32)	
3	46 (74)	59 (64)		8 (58)	24 (77)		12 (67)	15 (48)	
4	5 (8)	13 (14)		3 (21)	5 (16)		3 (17)	3 (10)	
Clinical T stage									
1	1 (1)	0 (0)	0.18	0 (0)	1 (3)	0.81	1 (5)	4 (13)	0.16
2	11 (18)	24 (26)		3 (21)	5 (16)		3 (17)	11 (35)	
3	24 (39)	39 (42)		3 (21)	9 (29)		8 (45)	8 (26)	
4	26 (42)	29 (32)		8 (58)	16 (52)		6 (33)	8 (26)	
Clinical N stage									
0	53 (85)	81 (88)	0.71	12 (86)	27 (87)	0.87	15 (83)	24 (77)	0.67
1	7 (11)	5 (5)		0 (0)	1 (3)		2 (11)	6 (19)	
2	1 (2)	5 (5)		2 (14)	3 (10)		1 (5)	1 (4)	
3	1 (2)	1 (2)		0 (0)	0 (0)		0 (0)	0 (0)	
Clinical M stage									
0	60 (97)	84 (91)	0.31	13 (93)	29 (94)	1	17 (95)	28 (90)	1
1	2 (3)	8 (9)		1 (7)	2 (6)		1 (5)	3 (10)	
Tumor location									
Cervical	1 (2)	3 (4)	0.92	0 (0)	0 (0)	0.82	0 (0)	3 (10)	0.17
Upper thoracic	17 (27)	23 (25)		2 (14)	3 (9)		4 (22)	10 (32)	
Middle thoracic	27 (44)	38 (41)		6 (43)	12 (39)		11 (61)	9 (29)	
Lower thoracic	17 (27)	28 (30)		6 (43)	16 (52)		3 (17)	9 (29)	
General type									
Medullary type	35 (56)	56 (61)	0.42	7 (50)	17 (55)	0.53	11 (61)	19 (61)	0.51
Fungating type	12 (19)	9 (10)		1 (7)	0		3 (17)	3 (10)	
Ulcerative type	12 (19)	21 (23)		6 (43)	12 (39)		3 (17)	3 (10)	
Constrictive type	3 (6)	6 (6)		0	2 (6)		1 (5)	6 (19)	
Tobacco use									
No	39 (63)	47 (51)	0.15	10 (71)	20 (65)	0.91	7 (39)	20 (65)	0.08
Yes	23 (37)	45 (49)		4 (29)	11 (35)		11 (61)	11 (35)	
Alcohol use									
No	51 (82)	72 (78)	0.54	10 (71)	23 (74)	1	12 (67)	29 (94)	0.04
Yes	11 (18)	20 (22)		4 (29)	8 (26)		6 (33)	2 (6)	

Analyzing the patients' characteristics by univariate logistic regression showed no associations with radiotherapy response, including sex, age, maximum diameter of tumor before radiotherapy, clinical tumor stage, clinical node stage, clinical metastatic stage, clinical stage, general type, tumor location, alcohol use, and tobacco use in each cohort (*P* > *0.05*) (Supplementary Table S1).

### Machine learning radiomics models using random forest for predicting response to radiotherapy

**Figure 3:**
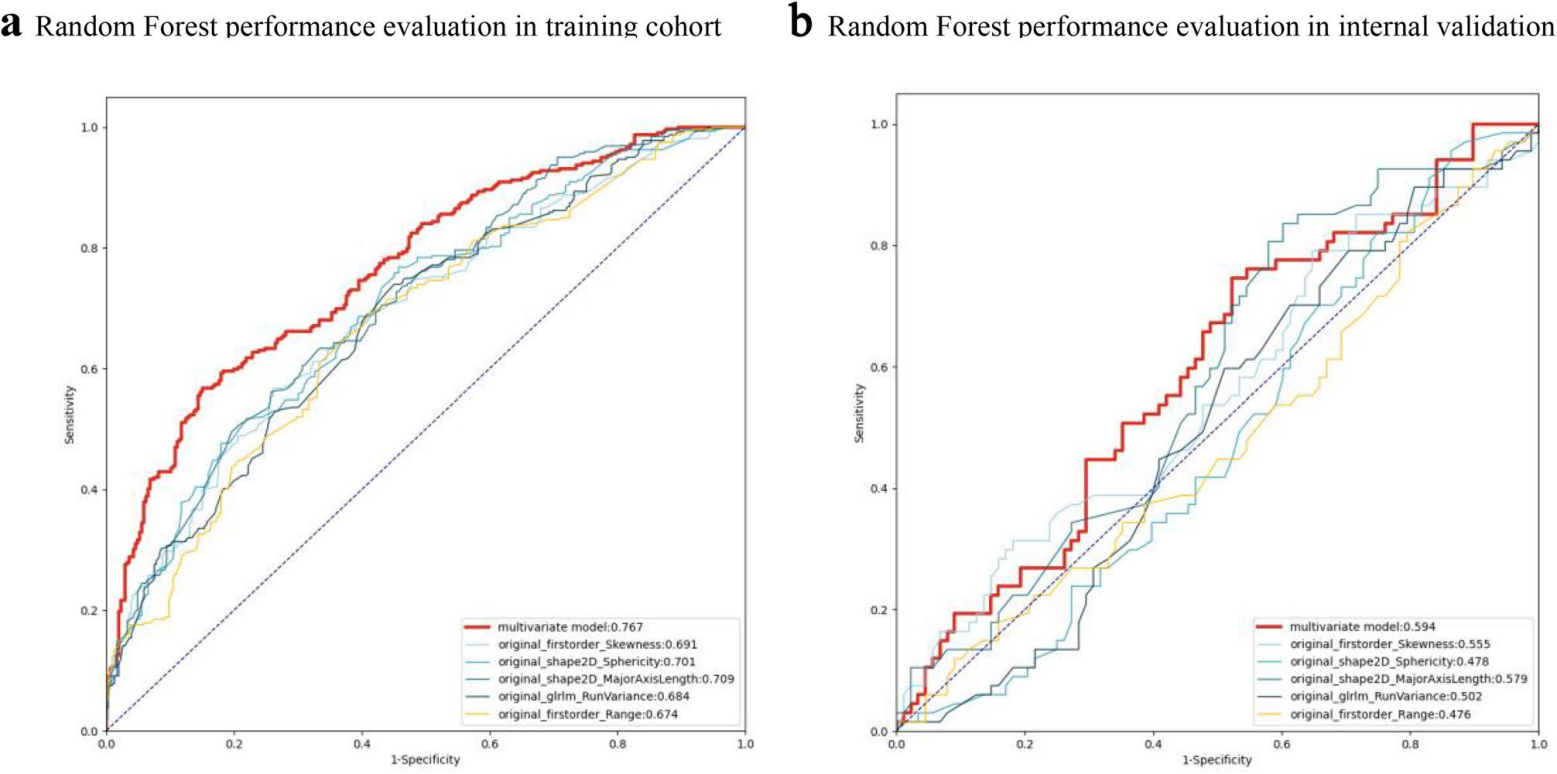
(**a**,**b**) The random forest model’s receiver operating characteristic curves of radiomic signatures in the primary cohort and internal cohort. The thick red line illustrates the ROC curve of the random forest model using all top 10 features, while other lines represent random forest models using the top 5 features separately.

Four machine learning classifiers were used to construct radiomics models, including linear regression, SVM with linear kernel, SVM with radial basis kernel, and random forest models. The ten most predictive features identified by the random forest algorithm were selected to train the classifier. Compared with the performance outcomes of each classifier, the random forest model showed the highest AUCs in the training and internal validation cohorts, which were 0.767 (95% CI, 0.734–0.790) and 0.594 (95% CI, 0.562–0.631), respectively. The SVM with a linear kernel achieved an AUC of 0.561 (95% CI, 0.530–0.594) in the internal validation cohort, while the SVM with a radial basis kernel model achieved an AUC of 0.539 (95% CI, 0.510–0.564) in the internal cohort. The AUC of the linear regression model in the internal validation cohort was 0.589 (95% CI, 0.561–0.646) (Supplementary Fig. S3a–c). Then, to compare the performance of combined features model with independent feature model, we used the five most predictive features of combined model to train the model individually by random forest. The performance of each independent feature in the radiomics model showed a lower AUC than that of the combined features in the same radiomics model (Fig. 3a and b, Supplementary Table S2).

### A deep learning radiomics model using pretrained ResNet50 and a channel attention mechanism for predicting response to radiotherapy

**Figure 4:**
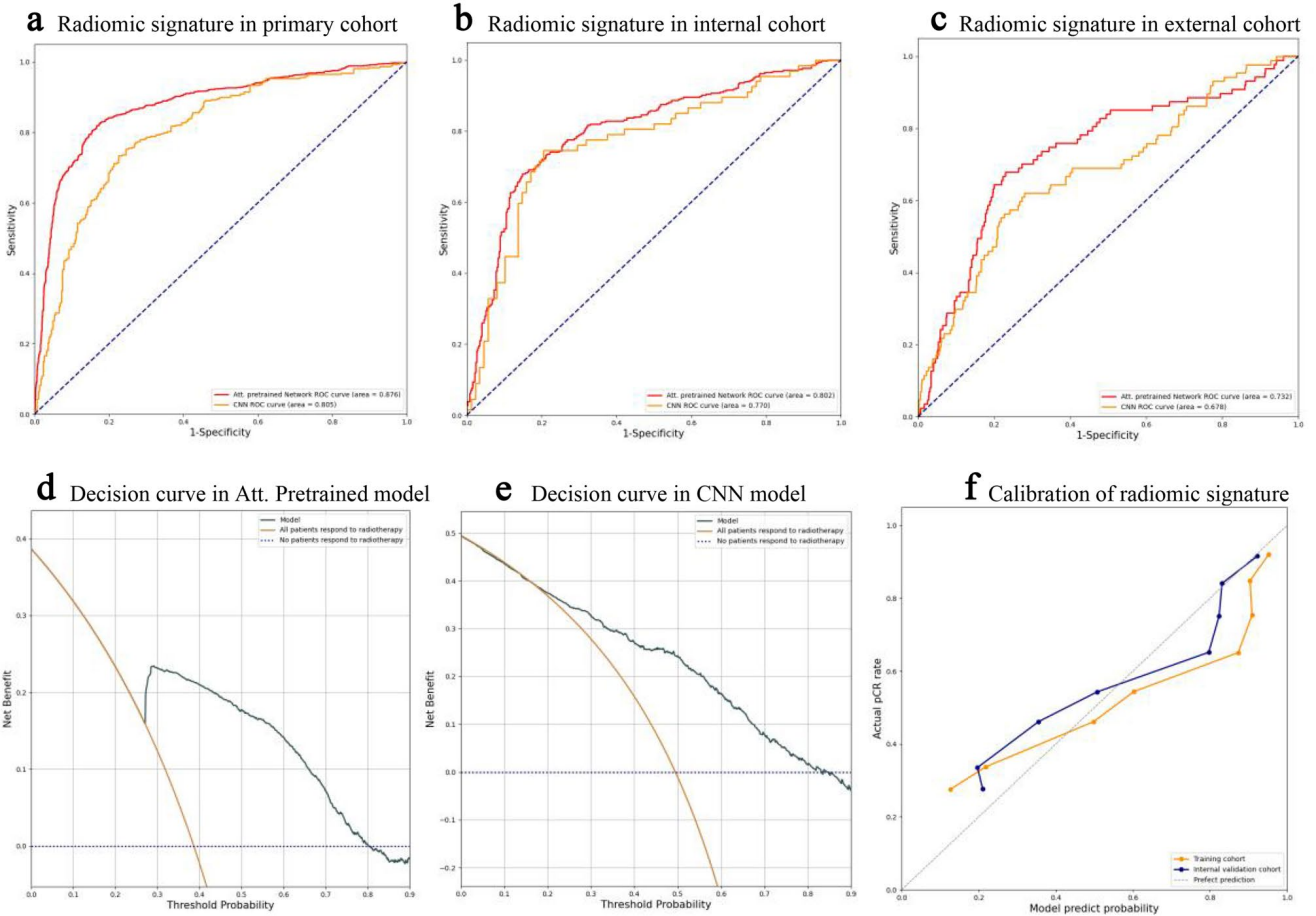
(**a**,**b**) and (**c**), Receiver operating characteristic curves of radiomic signatures in the primary cohort (**a**), internal cohort (**b**), and external cohort (**c**), which illustrate the comparison between Att. pretrained network and CNN model. D and E, Decision Curve of Att. Pretrained model (**d**) and CNN model (**e**). The brown line represents all patients who responded to radiotherapy, while the navy blue dotted line represents patients who did not respond to radiotherapy. (**f**), The plot illustrates the calibration of the radiomic signature in Att. Pretrained model. The dotted line indicates the ideal model. The orange line represents the calibration curve of the primary cohort, and the blue line represents the internal cohort.

The deep learning radiomics model constructed of pretrained ResNet50 and the channel attention mechanism outperformed all other methods in predicting radiotherapy response. The model achieved AUCs of 0 0.876 (95% CI 0.853–0.895), 0.802 (95% CI 0.775–0.837), and 0.732 (95% CI 0.672–0.797) in primary, internal and external cohorts, while the model trained by a CNN from scratch achieved AUCs of 0.805(95% CI 0.774–0.830), 0.770(95% CI 0.729–0.802), and 0.678(95% CI 0.619–0.738), respectively (Fig. 4a–c). This result indicated that the features of CT were feasible for constructing a reliable prognostic radiomics model. In comparing the performance of the machine learning radiomics models, the CNN radiomics models showed higher AUCs in all three cohorts, which revealed that the deep learning radiomics models, without decreasing data dimensions and removing redundant features, improved the performance of the radiomics model. The process of dimensionality reduction of radiomics features may lead to a lack of perspective information.

To estimate the predictive stability of the radiomics models and evaluate the benefits in clinical applications, decision curves and calibration curve were used to evaluate the performance of two neural network models. The decision curves showed that with the consideration of preferences of decision, using Att. Pretrained model will obtain more benefits than using CNN model in clinical practice (Fig. 4d, e). A calibration curve was applied to the pretrained ResNet50 model in the primary and internal cohorts (Fig. 4f), which showed a high degree of agreement between the predicted and actual results of the model.

## Discussion

Radiotherapy is considered one of the most crucial treatments for ESCC patients^36^. The construction of models for predicting the response to radiotherapy is significantly instructive for individualized precision treatment^37^. What makes radiation response prediction difficult in ESCC is the lack of predictive molecular markers of radiation sensitivity^38^. Moreover, the traditional clinical characteristics and general type of ESCC showed limited correlation with the response to radiotherapy. Thus, an available model that can efficiently predict the response to radiotherapy in patients with ESCC needs to be developed. Recently, radiomics signature models based on AI have been applied to different areas and have shown incredible performance in predicting the response to radiotherapy^39–43^. Here, we aimed to design a pretreatment CT-based radiomics model for radiotherapy response prediction in patients with ESCC, which can cover the shortage of predictive molecular markers.

Machining learning and deep learning models have been widely used in radiomics research^44–46^. In a previous study, traditional machining learning algorithms, such as random forest and SVM, were mentioned more frequently than deep learning algorithms due to the limitation of the population of cohorts^17,47^. Moreover, end-to-end algorithms in deep learning have begun to be used in cancer research in recent years, not only using radiographic images but also using histopathological images^48,49^. Screening the reproducibility of features, which is considered an indispensable part of reducing the overfitting of traditional machine learning radiomic models, seems to improve the performance of the radiomics model. In contrast, end-to-end algorithms do not need to reduce the dimensionality and aim to make full use of all image information to draw conclusions^50^. However, the superiority of these two types of algorithms has not been compared to predict radiotherapy response. To determine which of these two algorithms can construct a more effective radiomics model, in our study, both algorithms were used to construct radiomics models in the same ESCC patient cohorts. The results implied that whether in the training, internal validation or external validation cohorts, the radiomics model constructed by the end-to-end deep learning algorithm showed better performance. This suggests that end-to-end deep learning algorithms should receive more attention in subsequent radiomics studies. Moreover, the CNN model from scratch and the CNN pretrained model were also compared in our studies. Although the use of pretrained neural network models has become an increasingly mainstream choice in recent research, pretrained and models from scratch have rarely been compared in radiomics studies^51^. Our studies showed some evidence regarding pretrained models. Meanwhile, recent studies reported that the channel attention mechanism could significantly improve the performance of neural network models. In our study, the channel attention layer significantly improved the convergence difficulty caused by too many channels in the pretrained model and improved the performance of the model by nearly 3% in the external validation cohort.

Recently, advances in radiogenomics have shown that radiomics signatures have distinct correlations with gene expression patterns^37^. The radiomics signatures driven by different pathways involved in immune regulation, tumor proliferation, treatment responses and cellular functions further explain the biological basis of radiomics^52^. This result suggests that we can reflect intratumoral heterogeneity, to a certain extent, by constructing a radiomics model. Compared with traditional clinical characteristics, radiomics features better predict the treatment response to radiotherapy. Therefore, we hypothesize that end-to-end algorithms' overall utilization of CT image information may reflect tumor heterogeneity. The difference in extracting image information may cause an apparent discrepancy between these two different algorithms. However, these theories have not been elucidated by radiogenomics and multiomics studies.

Our retrospective study was limited to temporal discontinuities in the included patients. Although our study minimized differences in imaging data by using a standardizing process, the differences in CT equipment between each period and institution may lead to bias in collecting imaging data. To our knowledge, this study is the first multicenter study of radiomics in nonsurgical ESCC patients. However, the two institutions of our study are located in the same province, and the number of patients is limited. A large patient population of other regions is still needed to evaluate the extrapolation of the model. Finally, our study only analyzed the 2D radiomics phenotype and clinical characteristics due to the limitation of cohorts, and the 3D radiomics phenotype did not show good performance in our model. Therefore, the 3D radiomics phenomenon still needs to be explored in the next step. It is also necessary to combine other omics to further reveal the biological significance of radiomics.

This is the first multicenter radiomics study to develop an Att. Resnet50 pretrained network radiomics model in patients with advanced ESCC. It enables clinical decision-making, relying not only on the clinical doctors' experience but also on an objective basis. The effective prediction of radiotherapy provides these patients with reasonable individualized and precise treatment options, as well as timely alternative curative-intent treatment approaches to prevent any unnecessary side effects of radiotherapy and improve the quality of life and survival outcomes of advanced ESCC patients. In addition, our study uses existing routine diagnostic CT imaging, which does not add additional financial burden to patients. At the same time, the Att. Resnet50 pretrained network radiomics model does not require standardized extraction of radiomics signatures, which can be more convenient in clinical use for oncologists to predict the radiotherapy response during diagnosis.

## Conclusion

Developing an Att. Resnet50 pretrained network radiomics model for predicting the response to radiotherapy in patients with advanced ESCC can not only help oncologists formulate effective individualized radiotherapy plans promptly and guide clinical decision-making but also complement the lack of molecular markers for predicting radiosensitivity. It is hoped that our study can be included in the radiomics database of ESCC and be considered a baseline study of radiomics in advanced ESCC. The model has the potential to apply to other medical image classification tasks.

## Supplementary Information

Supplementary Information.

## Data Availability

The data sets generated and/or analyzed during the current study are available from the corresponding author on reasonable request.Code of our full approach are publicly available from Github (https://github.com/lqawakeme/RRAESCC).
