# Supplementary material for: An artificial neural network-based radiomics model for predicting the radiotherapy response of advanced esophageal squamous cell carcinoma patients: a multicenter study
Source: Sci Rep. 2023 May 29;13:8673. doi: 10.1038/s41598-023-35556-z (PMC10226996; doi:10.1038/s41598-023-35556-z)
Supplement: Supplementary file 1 — Supplementary Information. [file 41598_2023_35556_MOESM1_ESM.docx]

An Artificial Neural Network-Based Radiomics Model for Predicting the Radiotherapy Response of Advanced Esophageal Squamous Cell Carcinoma Patients: A Multicenter Study

Yuchen Xie^1^; Qiang Liu^2^; Chao Ji^1^; Yuchen Sun^1^; Shuliang Zhang^1^; Mingyu Hua^1^; Xueting Liu^1^; Shupei Pan^3^; Weibin Hu^1^; Yanfang Ma^1^; Ying Wang^1^ and Xiaozhi Zhang^1^


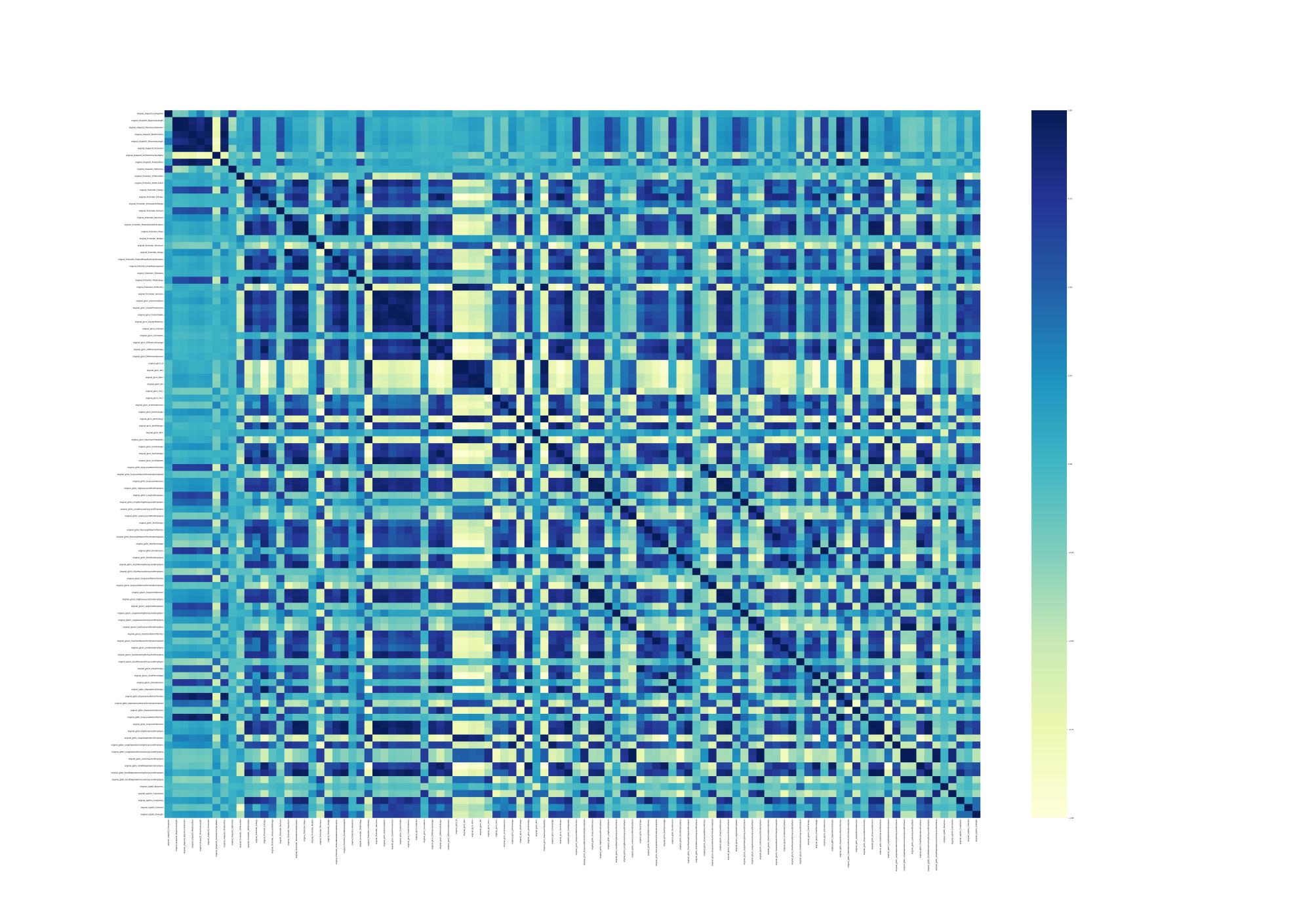


Supplementary Figure S1, Heat-map of the correlation coefficient between each pair of features. Python software version 3.8 (Python) was used for graphic depiction.


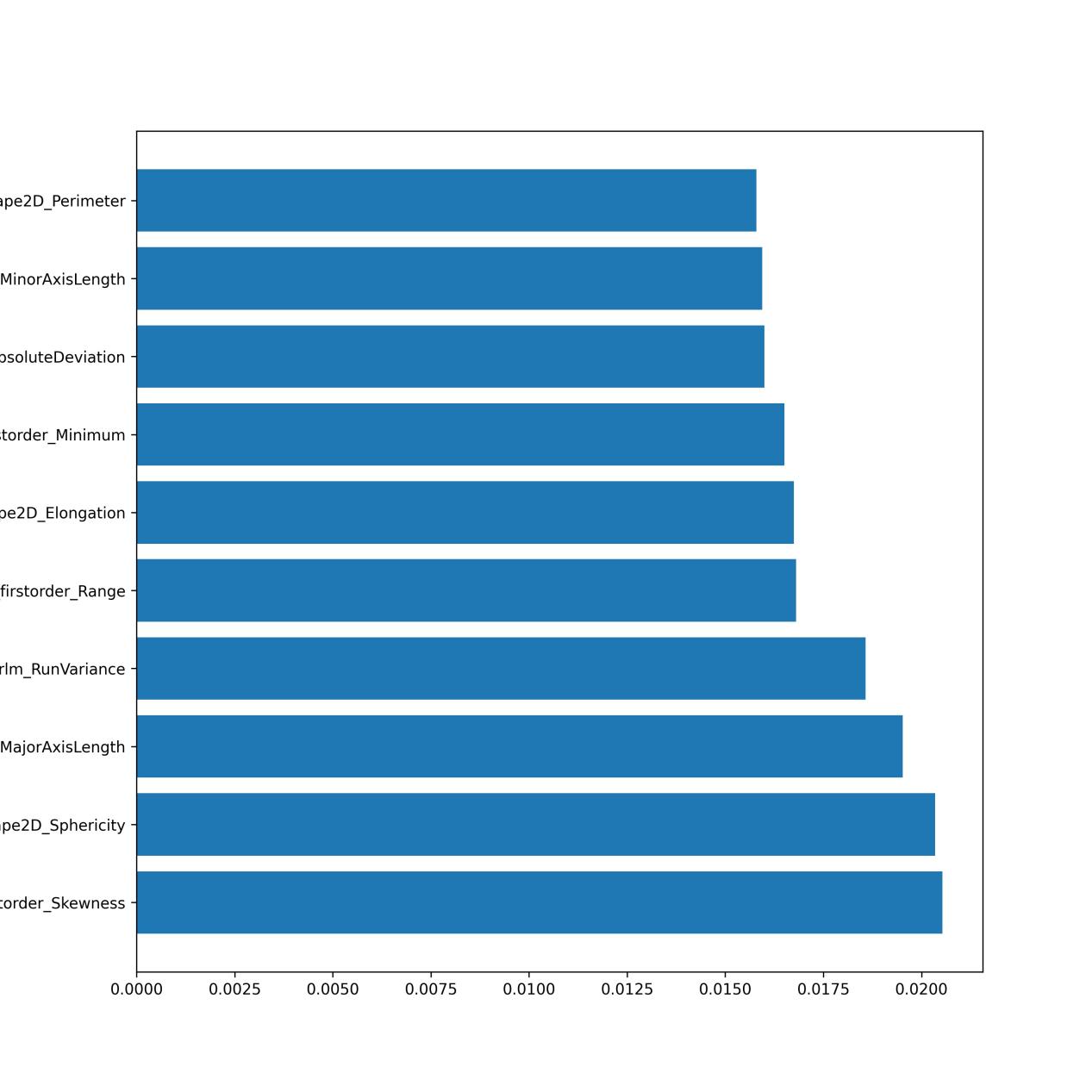


Supplementary Figure S2, Histogram of features that do not belong to the same correlation group among all features and have the best predictive power. Python software version 3.8 (Python) was used for graphic depiction.


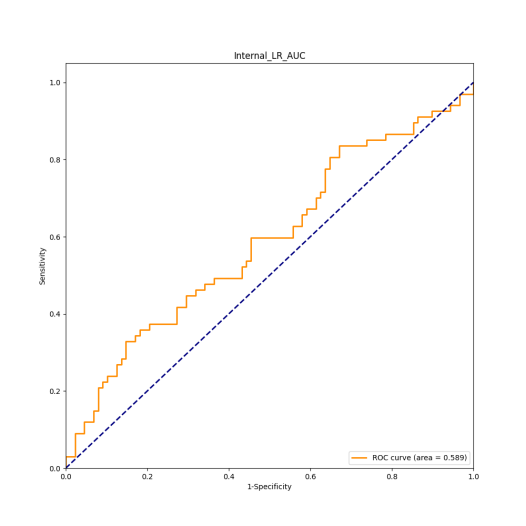

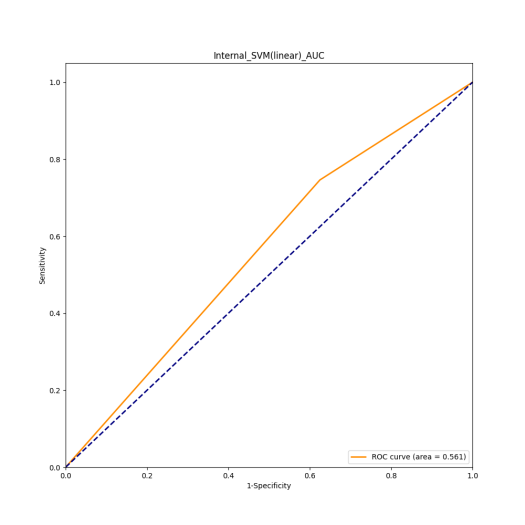

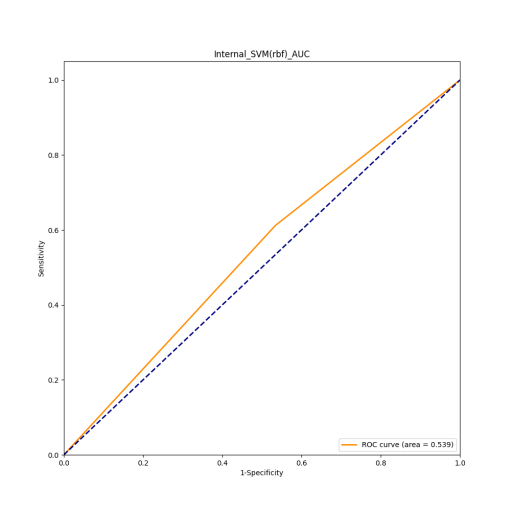


**a b c**

Supplementary Figure S3, a AUC curve of SVM model with rbf kernel in internal validation cohort; b AUC curve of SVM model with linear kernel in internal validation cohort; c AUC curve of Linear Regression model in internal validation cohort.

Supplementary Table S1, The univariate analysis of the association with clinical characteristics and radiotherapy reponse.

| Characteristics | OR | 95%CI | | *P* |
| --- | --- | --- | --- | --- |
|  |  | Upper limit | Lower limit |  |
| Sex | 1.01 | 0.43 | 2.37 | 0.98 |
| Age | 1.00 | 0.96 | 1.04 | 0.93 |
| Maximum diameter of tumor before radiotherapy | 0.94 | 0.85 | 1.05 | 0.26 |
| Clinical tumor stage | 0.72 | 0.30 | 1.75 | 0.47 |
| Clinical node stage | 0.70 | 0.31 | 1.57 | 0.38 |
| Clinical metastatic stage | 1.49 | 0.22 | 10.03 | 0.68 |
| Clinical stage | 2.25 | 0.68 | 7.5 | 0.19 |
| General type | 0.99 | 0.70 | 1.41 | 0.95 |
| Tumor location | 0.94 | 0.62 | 1.45 | 0.79 |
| Alcohol use | 1.09 | 0.39 | 3.06 | 0.88 |
| Tobacco use | 0.56 | 0.23 | 1.36 | 0.20 |

Supplementary Table S2, The comparison of the performance of combined features model with 5 most predictive features independent model by random forest in training and internal validation cohorts.

| features | AUC | |
| --- | --- | --- |
|  | Training cohort | Internal validation cohort |
| multivariate model | 0.767 | 0.594 |
| original_firstorder_Skewness | 0.691 | 0.555 |
| original_shape2D_Sphericity | 0.701 | 0.478 |
| original_shape2D_MajorAxisLength | 0.709 | 0.579 |
| original_glrlm_RunVariance | 0.684 | 0.502 |
| original_firstorder_Range | 0.674 | 0.476 |
